# Feature ranking based on synergy networks to identify prognostic markers in DPT-1

**DOI:** 10.1186/1687-4153-2013-12

**Published:** 2013-09-19

**Authors:** Amin Ahmadi Adl, Xiaoning Qian, Ping Xu, Kendra Vehik, Jeffrey P Krischer

**Affiliations:** 1 Department of Computer Science and Engineering, University of South Florida, Tampa, FL, 33620, USA; 2 Department of Electrical & Computer Engineering, Texas A&M University, College Station, TX, 77843, USA; 3 Department of Pediatrics, College of Medicine, University of South Florida, Tampa, FL, 33613, USA

**Keywords:** DPT-1, Type 1 diabetes, Biomarker identification, Interaction, Synergy network, Feature ranking

## Abstract

Interaction among different risk factors plays an important role in the development and progress of complex disease, such as diabetes. However, traditional epidemiological methods often focus on analyzing individual or a few ‘essential’ risk factors, hopefully to obtain some insights into the etiology of complex disease. In this paper, we propose a systematic framework for risk factor analysis based on a synergy network, which enables better identification of potential risk factors that may serve as prognostic markers for complex disease. A spectral approximate algorithm is derived to solve this network optimization problem, which leads to a new network-based feature ranking method that improves the traditional feature ranking by taking into account the pairwise synergistic interactions among risk factors in addition to their individual predictive power. We first evaluate the performance of our method based on simulated datasets, and then, we use our method to study immunologic and metabolic indices based on the Diabetes Prevention Trial-Type 1 (DPT-1) study that may provide prognostic and diagnostic information regarding the development of type 1 diabetes. The performance comparison based on both simulated and DPT-1 datasets demonstrates that our network-based ranking method provides prognostic markers with higher predictive power than traditional analysis based on individual factors.

## Introduction

Type 1 diabetes (T1D) is an autoimmune disorder and one of the common pediatric diseases with a diverse pathogenesis, clinical phenotype, and outcome [1]. Despite the emergence of T1D as a global issue with a steady increase in incidence worldwide over the past decade [2], the etiology of T1D is still not fully understood. Recent studies, including the Diabetes Prevention Trial-Type 1 (DPT-1) [3], have suggested that this complex disease has multiple risk factors, including genetic predisposition, diet, viruses, and geography in addition to autoimmunity [1,4-7]. The previous epidemiology studies mostly focus on studying hypotheses regarding individual risk factors, which have obtained important initial understanding, including the predisposing roles from genetic markers such as human leukocyte antigens [5]. However, traditional hypothesis-driven approaches focusing on ‘essential’ factors may not be sufficient for fully understanding T1D [6]. With large-scale perspective studies such as DPT-1, we believe that data-driven investigation considering all candidate factors with their interactions can serve as a critical complement for previous hypothesis-driven research.

Data-driven methods have been proven to be useful in both identifying probable mechanisms involved in disease and providing accurate biomarkers for early prediction [8,9]. However, as shown in genome-wide association studies (GWAS), single marker analysis is not sufficient for genetic studies of complex diseases [10,11]. In order to better explain the missing heritability of complex disease through analyzing high-dimensional genotype data, several methods have been proposed to take into account the interactive effect among single-nucleotide polymorphisms as well as multiple genes in GWAS and other -omic data analysis [12-14]. In this work, we propose a network-based mathematical model for systematically analyzing candidate risk factors for disease. We consider that the individual effect and interactions from potential risk factors are all manifested as statistical associations with the disease outcome. Based on this, we construct a synergy network which integrates both the individual and synergistic interactive effects of factors in one single graph structure. We then propose a novel algorithm based on this synergy network to identify biomarkers for early prediction of disease. Specifically, we verify the effectiveness of our method using simulated case-control datasets. With such validated results, we apply our method to identify biomarkers for prognosis of T1D from measured immunologic and metabolic indices in DPT-1. The performance of the identified markers is then compared to the performance of traditional forward feature selection which only considers the individual statistical association with outcome. Our comprehensive results show that our network-based method identifies better biomarkers with better predictive performance.

## Methods

Feature selection approaches are commonly used to identify biomarkers by finding a subset of biomedical measurements with high predictive power with respect to disease outcome [15-17]. As it is computationally very expensive to exhaustively search for the best subset of variables, these methods mostly rely on heuristic approaches. Filtering variables based on their individual effect on disease outcome has been a common practice in biomedical research. Heuristic approaches based on filtering have been successful in identifying biomarkers with strong individual effects. However, they may miss variables with weak individual effects but having synergistic interactive effects that produce high predictive accuracy [15,17]. To avoid missing these critical variables with high synergistic effects on outcome, we propose a new approach which takes into account both individual and synergistic interactive effects. In our approach, we first construct a synergy network based on the individual and synergistic effects of all the observed variables. Then, we solve the problem of finding the best subnetwork by an efficient graph spectral algorithm which leads to a novel feature ranking that improves the traditional ranking by taking into account the interaction among variables. Finally, we use this feature ranking together with traditional forward feature selection to achieve the final set of biomarkers.

### Synergy network

To construct the synergy network, we need to measure the individual predictive power of all variables together with their pairwise synergistic power. One natural way to measure both individual and synergistic powers is to use a logistic regression model. In order to measure the individual power of variable *v*_*i*_, we can learn the following logistic model log(*g*/(1−*g*))=*α*_0_+*α*_1_*v*_*i*_ in which *g* is the probability *p*(*y*=1|*v*_*i*_), where *y* denotes the disease outcome of interest. After fitting this model to the given data, the magnitude of the coefficient *α*_1_ measures the individual power of *v*_*i*_. To make sure that the measurements for different variables are with the same unit and comparable to each other, we use − log(*p*_*i*_) as the individual power of variable *v*_*i*_, in which *p*_*i*_ is the coefficient *p*-value for *α*_1_ and measures the statistical significance of the individual power of *v*_*i*_. Similarly, in order to measure the synergistic predictive power between two variables *v*_*i*_ and *v*_*j*_, we fit the following logistic model log(*g*/(1−*g*))=*α*_0_+*α*_1_*v*_*i*_+*α*_2_*v*_*j*_+*βv*_*i*_*v*_*j*_ (where *g*=*p*(*y*=1|*v*_*i*_,*v*_*j*_)) to data and consider − log(*p*_*ij*_) as the synergistic power of variables *v*_*i*_ and *v*_*j*_, in which *p*_*ij*_ is the coefficient *p*-value of *β*. With that, we construct the synergy network which can be represented by a graph *G*(*V*,*E*). In this synergy network, *V* is the set of nodes corresponding to all the variables, and each *v*_*i*_∈*V* has the node weight *f*(*v*_*i*_) equal to − log(*p*_*i*_); *E* is the set of edges (*v*_*i*_,*v*_*j*_) with the edge weight *s*(*v*_*i*_,*v*_*j*_) equal to − log(*p*_*ij*_).

### Finding subnetworks for biomarker identification

As explained, the synergy network integrates both individual and synergistic powers of candidate risk factors in a single graph structure. Similar to the traditional problem of feature selection, here we are looking for subsets of risk factors or subnetworks in the synergy network, with the highest possible discriminative power regarding disease outcome *y*. To simplify the problem, we approximate the discriminative power of subnetworks by the summation of the node weights and edge weights induced in them. We note that this approximation is expected to perform better than traditional feature selection approaches based on only individual effects [16] due to the integration of synergistic effects in our synergy network. The biomarker identification problem is then reduced to solve the following optimization problem:

(1) $$\begin{array}{l} \underset{C\subseteq G}{\text{max}}\underset{v_{i}\in C}{\sum }f(v_{i})+\lambda \underset{v_{i},v_{j}\in C}{\sum }s(v_{i},v_{j}), \end{array}$$

where *C* denotes potential subnetworks and 0≤*λ*≤1 is a weighting coefficient between individual and synergistic effects. As both *f*(*v*_*i*_) and *s*(*v*_*i*_,*v*_*j*_) are nonnegative, the previous optimization problem has the degenerated solution to include all the risk factors in *C*. To overcome this problem, we further impose another constraint to restrict the size of selected subnetworks to have |*C*|≤*K*. This formulation is in fact the problem of finding a maximum weighted clique (MWCP) [18] which is a generalization of the classical maximum clique problem (MCP). As MCP is nondeterministically polynomial (NP)-hard [19], it can be easily shown that MWCP is NP-hard as well. Thus, our biomarker identification problem formulated in Equation 1 is also an NP-hard problem. Several approaches have been previously proposed to find the exact optimal solution of the problem by employing branch-and-bound techniques, but it is probable that exhaustive search over all possible subnetworks is needed [18]. In this paper, we propose a fast approximate algorithm for MWCP which also provides a ranked list of features based on both their individual and synergistic effects.

### Feature ranking by a graph spectral algorithm

We first rewrite the optimization problem given in Equation 1 as a quadratic integer programming problem as follows: For each node *v*_*i*_ in *G*, we consider an integer variable *x*_*i*_ which is equal to 1 if the node *v*_*i*_ is selected in the subnetwork *C* and is 0 otherwise. Using this variable, we can rewrite Equation 1 as ${\text{max}}_{\text{x}}={\left[\;x_{1},x_{2},\ldots x_{n}\right]}^{T}\sum_{i=1}^{n}f(v_{i})x_{i}^{2}+\lambda \sum_{i,j=1}^{n}s(v_{i},v_{j})x_{i}x_{j}$, where *n* is the number of feature nodes in *G*. We further define the matrix *M*_(*n*×*n*)_ with diagonal entries *M*_*i*,*i*_ equal to the individual power *f*(*v*_*i*_), and off-diagonal entries *M*_*i*,*j*_ equal to the synergistic power *λ*×*s*(*v*_*i*_,*v*_*j*_). We can rewrite the optimization problem for biomarker identification in the following matrix format:

(2) $$\begin{array}{l} \;\underset{x}{\text{max}}\;x^{T}Mx\;\\ \text{s.t.}\;\;x^{T}x\leq K;\\ \;x_{i}\in \{0,1\}, \end{array}$$

in which **x**= [ *x*_1_,⋯,*x*_*n*_]^*T*^ is a binary integer vector. In fact, the size constraint is equivalent to putting in a sparse penalty on **x** to select the smallest number of risk factors that have high predictive power. In order to solve this constrained quadratic integer programming problem, we develop a spectral approximate algorithm. We first relax the integer variable *x*_*i*_∈{0,1} to $x_{i}\in R$. Then, using Lagrangian relaxation, we can transform the original optimization problem given in Equation 2 to the following quadratic programming optimization problem:

(3) $$\underset{x}{\text{max}}\;x^{T}Mx+\alpha (K-x^{T}x),$$

where *α* is the Lagrangian multiplier. Based on the Karush-Kuhn-Tucker condition [20], the optimal solution of this relaxed quadratic programming problem has to (necessarily) satisfy the condition that the derivative of the relaxed objective function equals to 0:

(4) $$\begin{array}{l} \frac{\partial }{\partial x}\left[\right)x^{T}Mx+\alpha (K-x^{T}x)])=0. \end{array}$$

By straightforward algebraic manipulations, we can show that the potential solution **x**^∗^ has to satisfy *M***x**^∗^=*α***x**^∗^. Therefore, the relaxed solution **x**^∗^ to the MWCP is an eigenvector of the matrix *M*. Furthermore, we want the objective function **x**^∗*T*^*M***x**^∗^=*α***x**^∗*T*^**x**^∗^=*αK* to have the maximum value with **x**^∗^, which means that we want *α* to be as large as possible. Hence, the solution **x**^∗^ will be the eigenvector of *M* with the largest corresponding eigenvalue. Also given the relaxed solution **x**^∗^, for any *K*, the approximate solution to the original integer programming optimization problem is to take top *K* nodes with the largest corresponding magnitudes in **x**^∗^. This also shows that the candidate risk factors with larger magnitudes in **x**^∗^ are more desirable to be selected in the final subset of risk factors as potential prognostic biomarkers. Thus, we can use the absolute values in **x**^∗^ as a score to rank the risk factors. We note that *K* can be an arbitrary number without loss of generality, which will not affect our final ranking as the **x**^∗^ only depends on the matrix *M*. As one can see, the proposed method combines both individual power and synergistic power among all candidate risk factors into one single score that can be used to rank them.

## Biomarker identification using network-based spectral ranking

In order to select a subset of risk factors based on any ranking, a common approach is to use forward feature selection [16]. We replace the ranking step of the forward feature selection, which is only based on individual power, by our network-based spectral ranking which takes into account the interaction among factors as well. In forward feature selection, we sequentially add potential risk factors from the top of the ranked list to the current set of selected factors only if it improves the classification performance; otherwise, we move to the next factor in the ranked list. This procedure is repeated until we reach the end of the ranked list.

## Experiments and discussions

We evaluate the performance of our network-based biomarker identification based on both simulated datasets and datasets obtained from the DPT-1 study and compare it with the individual-based biomarker identification, which only considers individual effects. In order to properly estimate and compare the performance of biomarker identification methods, we perform an ‘embedded’ cross-validation procedure.

### Performance evaluation procedure

As explained earlier, our feature selection approach includes two steps: First, we construct a synergy network based on the given dataset and rank the candidate risk factors using our spectral algorithm. Second, we use the ranked list of factors obtained in the first step to perform a forward feature selection [16]. To make sure that we do not overestimate the performance of our biomarker identification approach, we perform the following embedded cross-validation procedure: Similar to the regular ten-fold cross validation, we first randomly divide the dataset into ten folds, within which one fold is used as the *testing set* to test the performance and the remaining nine folds are used as the *training set* to select biomarkers and learn the classifier. In order to select biomarkers based on the training set, we first use all the data points in the training set to construct a synergy network and perform our spectral algorithm to obtain the ranked list. Then, using the ranked list, we perform a forward feature selection method to select the best performing set of biomarkers. In the forward feature selection method, we sequentially add candidate factors to the current feature set (starting with an empty set), if it improves the classification performance; otherwise, we move to the next factor in the ranked list. To evaluate the performance of a set of potential risk factors during forward feature selection, we use another standard ten-fold cross validation in which we further divide the training set into ten folds, nine of which are used to train the classifier and the remaining is used to test the performance. After performing the forward feature selection and identifying the biomarkers, we learn a classifier based on the *training dataset* using those selected features and compute the performance based on the testing set. During our performance evaluation procedure, we adopt the MATLAB implementation of quadratic discriminant analysis as the classifier [21] to make sure that the pairwise interaction among risk factors is taken into account by the classifier. To measure the performance of any classifier in our performance evaluation procedure, in addition to the accuracy, we also compute the area under the ROC curve (AUC) which is a more reliable measure of prediction performance [22] in our experiments. When we use accuracy as the performance measure during forward feature selection, the identified biomarkers are optimized to provide better accuracy. We also take AUC as the performance measure for forward feature selection so that the biomarkers are optimized to provide better AUC. This two sets of biomarkers are not necessarily the same, especially with unbalanced datasets, as they are supposed to optimize for different criteria. Thus, for each dataset, we have two sets of results: one based on accuracy and one based on AUC.

### Performance comparison based on the simulated datasets

We simulate a case-control disease model, in which the outcome *y* (disease) follows a Bernoulli distribution with the success parameter equal to *p*(*y*=1|**v**) given the input variables **v**. We first simulate 30 random variables as input variables **v**=[ *v*_1_,*v*_2_,…,*v*_30_]^*T*^. From all 435 potential pairs of these randomly simulated variables, ten of them are randomly selected to have synergistic effects with respect to the outcome. Based on this, we follow the following logistic model to simulate the disease outcome *y*:

(5) $$\begin{array}{l} \log\left(\frac{p(\;y=1|v)}{1-p(\;y=1|v)}\right)=\alpha_{0}+\overset{30}{\underset{i=1}{\sum }}\alpha_{i}v_{i}+\underset{i\neq j}{\sum }\beta_{\text{ij}}v_{i}v_{j}. \end{array}$$

In this logistic model, the magnitude of each individual coefficient *α*_*i*_ determines the individual effect of the corresponding variable *v*_*i*_ on outcome *y*, and the magnitude of the interaction coefficient *β*_*ij*_ determines the amount of synergistic effect of two variables *v*_*i*_ and *v*_*j*_ on the outcome. To obtain the previously described case-control data, we simulate 30 random features with each variable *v*_*i*_ following a mixture-of-Gaussian distribution with equally weighted (mixture parameters equal to 0.5) Gaussian distributions with the same variance of 1.0 and the means equal to −1.0 and 1.0, respectively. For 435 interaction coefficients *β*_*ij*_, we randomly set 425 of them to zero, and the values of the other ten are drawn from the standard normal distribution (mean 0.0 and variance 1.0). We also set all the individual coefficients *α*_*i*_ to zero which means that there is no feature with significant individual effect. To simulate the outcome *y*, we first compute the probability *p*(*y*=1|**v**) based on the previous logistic model (Equation 5). Then, we generate the value for *y* from a Bernoulli distribution with the success parameter equal to *p*(*y*=1|**v**). We have generated 20 of such case-control datasets with 200 data samples in each set for the performance evaluation of our method. In order to make sure that our performance comparison results are independent of how we set the values of these coefficients, each of these 20 datasets is simulated with different random values for coefficients *β*_*ij*_.

**Figure 1:**
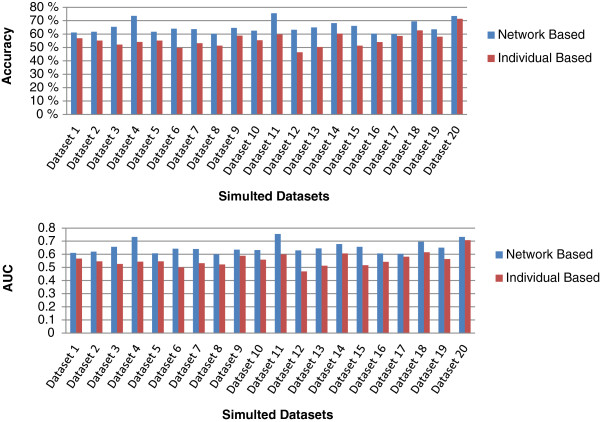
**Performance comparison between individual-based and network-based ranking for 20 simulated datasets.** Note that we have weak individual effects and significant synergistic effects in this ensemble of datasets.

To demonstrate the advantage of our network-based feature ranking, we compare the performance of our ranking with the traditional individual-based feature ranking. We use our embedded cross-validation procedure to evaluate the performance of both network-based ranking and individual-based ranking. We repeat the embedded cross validation 100 times for both individual- and network-based rankings and calculate the average accuracy and AUC for both methods. The performance comparison for our 20 simulated datasets is shown in Figure 1. The average accuracy and average AUC of our network-based method among 20 datasets are 65.17*%* and 0.6518, respectively, compared to 55.74*%* and 0.5577 obtained by individual-based ranking. As expected, the performance of our network-based ranking is significantly better than individual-based ranking. This clearly shows that filtering methods based on individual ranking are unable to capture those risk factors with synergistic effects but weak individual effects, which are critical biomarkers for better prediction.

**Figure 2:**
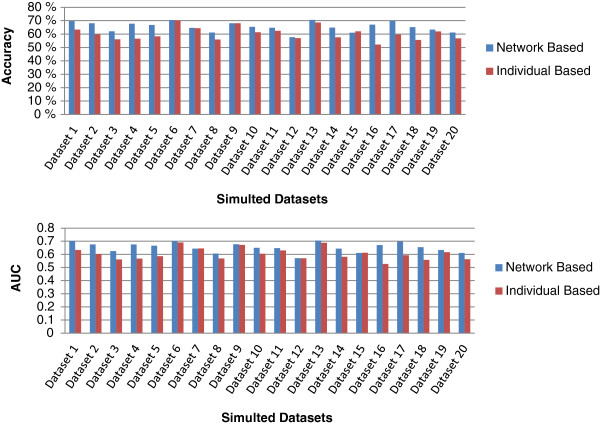
**Performance comparison between individual-based and network-based ranking for the other 20 simulated datasets.** Note that we have both significant individual effects and significant synergistic effects in this ensemble.

In order to further show that our network-based method does not only bias toward risk factors with only synergistic effects, we further check the performance of our network-based ranking when there are risk factors with significant individual effects in the case-control disease model. We use the same logistic regression model in Equation 5 where in addition to 10 nonzero interaction coefficients *β*_*ij*_, we also have five random nonzero individual coefficients *α*_*i*_ (*α*_0_ is set to zero as well). The values for those nonzero *α*_*i*_ are also drawn from a standard normal distribution. We have also generated 20 datasets of this new model, each with 200 samples. Similar to the previous 20 datasets, each of these 20 datasets is simulated with different random values for coefficients *α*_*i*_ and *β*_*ij*_. The performance evaluation results based on these 20 new simulated datasets are shown in Figure 2. The average accuracy and average AUC obtained by our network-based method among these 20 new datasets are 65.47*%* and 0.6536, respectively, both of which are significantly higher than 60.38*%* and 0.6040 obtained by individual-based ranking. This shows that our network-based ranking consistently performs better than individual ranking even when there are features with significant individual effects.

Finally, in our simulation model, we always have *p*(*y*=1)=*p*(*y*=0), which is due to the symmetry of the logistic function, symmetry of distribution of all features, and symmetry of distribution of coefficients around zero. As a result, the datasets simulated from the model are balanced, i.e., they have almost the same number of case and control samples. Because of this, the accuracy and AUC performance measures are very similar for all of our simulated datasets which might not be the case for unbalanced datasets.

### Biomarker identification in DPT-1

DPT-1 was a study designed to determine if T1D can be prevented or delayed by preclinical intervention of insulin supplement. It focuses on first- and second-degree nondiabetic relatives of patients with T1D before the age of 45, since they have more than tenfold risk of developing T1D compared to the general population [3]. DPT-1 screened 103,391 subjects altogether and categorized them into four risk groups based on genetic susceptibility, age, the presence of autoantibodies (including islet cell autoantibodies (ICA), insulin autoantibodies (IAA), glutamic acid decarboxylase (GAD), insulinoma-associated protein 2 (ICA512)), and the change of metabolic markers during oral glucose tolerance test (OGTT) and IV glucose tolerance test (IVGTT). The 3,483 subjects positive for ICA were staged to quantify the projected 5-year risk of diabetes [7]. Our analysis focuses on the study for the ‘high risk’ and ‘intermediate risk’ groups [7-9], which contain 339 and 372 subjects, respectively. The subjects of each group were randomly divided into two roughly equal subgroups: one received parenteral or oral insulin supplement, while the other was assigned to the placebo arm of the study. In this paper, we focus on the subjects of the placebo group. We consider the placebo subgroups of both high-risk and intermediate-risk groups as a dataset for our data-driven analysis (analysis based on the treated group is provided in Additional file 1). The dataset contains the following 19 features from baseline characteristics in DPT-1, focusing on immunologic and metabolic markers. We have taken the available titer values for different autoantibodies, including ICA, IAA, GAD, ICA512, and micro-insulin autoantibodies. For metabolic indices, we have fasting glucose, glycated hemoglobin (HbA1c), fasting insulin, and first-phase insulin response (FPIR) from IVGTTs. Homeostasis model assessment of insulin resistance (HOMA-IR) and FPIR-to-HOMA-IR ratio are also computed as in [9]. From OGTTs, in addition to 2-h glucose and fasting glucose, we have collected blood samples for C-peptide measurements in the fasting state and then 30, 60, 90, and 120 min after oral glucose, from which we have computed peak C-peptide as the maximum point of all measurements and AUC C-peptide using the trapezoid rule. Furthermore, as age and body mass index (BMI) have been conjectured to be important confounding factors, we also include them in our set of features. We are interested in identifying the most predictive group of features as biomarkers from the above described candidates to predict the outcome which is the development of T1D at the end of the DPT-1 study. The dataset contains 356 subjects within which 133 subjects developed T1D at the end of the study.

To check the performance of our network-based biomarker identification for DPT-1, similar to simulated datasets, we repeat the embedded cross validation 100 times and use the average performance. In order to show the advantage of our network-based method, we also compute the performance of individual-based feature ranking. The results based on both accuracy and AUC measurements are given in Table 1. As one can see, both accuracy and AUC obtained by our network-based ranking are significantly higher than individual-based ranking with *p*-values of 6.8e −04 and 7.17e −11, respectively. The results obtained based on both simulated and DPT-1 dataset clearly show that our spectral network-based feature ranking provides biomarkers with significantly better predictive power than individual-based feature ranking. This also verifies our expectation that the integration of synergistic interaction among features provides biomarkers with higher prediction accuracies.

**Table 1:** Accuracy and AUC performance of network-based ranking and individual-based ranking based on the DPT-1 dataset

**Performancemeasure**	**Individual ranking**	**Network-based ranking**	***p*-value**
Accuracy	68.31%	69.14%	6.8e −04
AUC	0.6524	0.6724	7.17e −11

In each run of the embedded ten-fold cross validation procedure, we in fact have ten possibly different sets of selected features as we perform feature selection for each fold based on a different subset of training samples at each run of the cross-validation procedure. By repeating this procedure 100 times, we obtain 1,000 (100 ×10) different subsets of biomarkers. In order to report a single reliable set of biomarkers, we first compute the frequency of the appearance of each feature and then select the features that at least appeared in 40% of the 1,000 (i.e., 400) selected subsets. The single set of biomarkers based on both individual and network-based rankings is provided in Table 2. We have also evaluated the performance of those final biomarkers by 100 repeated ten-fold cross validations. Their corresponding accuracies and AUCs are also given in Table 2.

**Table 2:** Final sets of biomarkers and their corresponding accuracy and AUC performances for the DPT-1 dataset

**Performance**	**Individual**		**Network-based**		**Exhaustive**	
**Measure**	**ranking**		**ranking**		**search**	
Accuracy	2-h glucose, IAA, ICA512, peakC-peptide, AUC C-peptide	70.59%	2-h glucose, IAA, fasting glucose (IVGTT), ICA512, peak C-peptide, AUC C-peptide, FPIR-to-HOMA-IR ratio	73.40%	2-h glucose, AUC C-peptide, BMI, FPIR-to-HOMA-IR ratio, fasting insulin (IVGTT), HOMAIR, HbA1c, IAA, ICA512, peak C-peptide	73.48%
AUC	age, 2-h glucose, IAA, ICA512, peak C-peptide, AUC C-peptide	0.6779	2-h glucose, IAA, FPIR, fasting glucose (IVGTT), ICA512, peak C-peptide, AUCC-peptide, FPIR-to-HOMA-IR ratio	0.7154	2-h glucose, age, FPIR-to-HOMA-IR ratio, fasting glucose (IVGTT), IAA, peak C-peptide, weight	0.7227

Individual ranking, network-based ranking, and exhaustive search methods were used.

Note that, as mentioned previously, the features selected during the forward feature selection step of our biomarker identification method might vary when we optimize different performance measures. As a result, the final set of biomarkers when we use accuracy in our performance evaluation is different from the final set of biomarkers when we use AUC. The final set of biomarkers using both accuracy and AUC is reported; however, based on the fact that AUC measurement is more reliable than accuracy for unbalanced datasets, we believe that the final set of biomarkers obtained by AUC is more reliable.

**Figure 3:**
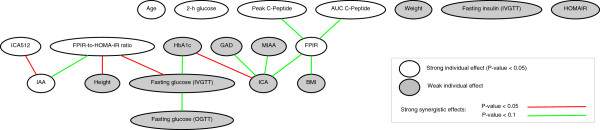
The average synergy network for the Placebo group in the DPT-1 dataset.

**Figure 4:**
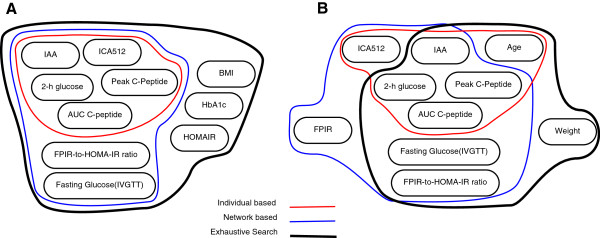
**Venn diagrams illustrating the identified biomarkers using different methods. (A)** Biomarkers identified by optimizing accuracy. **(B)** Biomarkers identified by optimizing AUC.

Due to the relatively small number of features in this study, it is feasible to perform an exhaustive search over all possible subsets of features to find the biomarker set with the best performance. We computed the AUC and accuracy of all 2^19^−1 possible subsets based on 100 repeated ten-fold cross validations. The best performing subsets together with their corresponding measured performances are also given in Table 2. The results in Table 2 clearly show that the network-based feature ranking method provides more predictive biomarkers than the individual-based feature ranking which are closer to the best performing biomarkers by exhaustive search. Furthermore, the average of 1,000 synergy networks obtained from 100 ×10 generation of synergy network in our embedded cross-validation procedure is provided in Figure 3. This synergy network shows that the nodes ‘FPIR-to-HOMA-IR ratio’, ‘fasting glucose (IVGTT)’, and ‘ICA’ are important nodes with high centrality in the average synergy network. From those three risk factors, FPIR-to-HOMA-IR ratio and fasting glucose (IVGTT) are also among the best biomarkers. This again verifies the effectiveness of our systematic network-based analysis in identifying important factors. Furthermore, as shown in Table 2, our network-based biomarker identification has successfully identified both of those important biomarkers, while the individual-based feature ranking has ignored them. We further provide in Figure 4 the Venn diagrams of selected biomarkers which show the intersection of biomarkers selected by different methods. As one can see, the intersection between biomarkers selected by our network-based ranking and best possible performing biomarkers is larger than the intersection between biomarkers selected by individual-based ranking and the best possible performing biomarkers.

## Conclusions

We have proposed a new feature ranking method that significantly improves the traditional feature ranking by considering the synergistic interaction among potential risk factors. The comprehensive results based on simulated datasets and the dataset from DPT-1 have shown that our network-based feature ranking can help identify more predictive biomarkers than traditional individual-based feature ranking. The set of final biomarkers identified for T1D may help find more predictive models for T1D which may provide early prediction of disease for timely treatment. Furthermore, the improvement obtained by our network-based data-driven method suggests that a more comprehensive systematic data-driven analysis of biomedical variables will be helpful for the better understanding of T1D etiology.

## Abbreviations

AUC: Area under ROC curve; BMI: Body mass index; DPT-1: Diabetes Prevention Trial-Type 1 (DPT-1) study; FPIR: First-phase insulin response; GAD: Glutamic acid decarboxylase; GWAS: Genome-wide association studies; HOMA-IR: Homeostasis model assessment of insulin resistance; IAA: Insulin autoantibodies; ICA: Islet cell autoantibodies; ICA512: Insulinoma-associated protein 2; IVGTTl: IV glucose tolerance test; MCP: Maximum clique problem; MWCP: Maximum weighted clique problem; NP: Nondeterministically polynomial; OGTT: Oral glucose tolerance test; ROC: Receiver operating characteristic; T1D: Type 1 diabetes.

## Competing interests

The authors declare that they have no competing interests.

## Authors’ contributions

AAA designed and implemented the algorithms, designed and carried out the experiments, analyzed the results, and drafted the manuscript. XQ conceived the study, designed the algorithms and the experiments, analyzed the results, and drafted the manuscript. PX, KV, and JPK helped analyze the results and drafted the manuscript. All authors read and approved the final manuscript.

## Supplementary Material

Additional file 1 **Supplementary material.** The results for network-based analysis based on the treated subjects from DPT-1 as well as a stability analysis for *λ* are provided in this file. Click here for file
