# Supplementary material for: Feature ranking based on synergy networks to identify prognostic markers in DPT-1
Source: EURASIP J Bioinform Syst Biol. 2013 Sep 19;2013(1):12. doi: 10.1186/1687-4153-2013-12 (PMC3849336; doi:10.1186/1687-4153-2013-12)
Supplement: Additional file 1 — Supplementary material. The results for network-based analysis based on the treated subjects from DPT-1 as well as a stability analysis for λ are provided in this file. [file 1687-4153-2013-12-S1.pdf]

Supplementary Material

Feature Ranking Based on Synergy Networks to Identify Prognostic Markers in DPT-1

Amin Ahmadi Adl[1] and Xiaoning Qian[1,2*] and Ping Xu[3] and Kendra Vehik[3] and Jeffrey P. Krischer[3]

[1]Dept. of Computer Science & Engineering, University of South Florida, Tampa, FL 33620
[2]Dept. of Electrical & Computer Engineering, Texas A&M University, College Station, TX 77843
[3]Dept. of Pediatrics, College of Medicine, University of South Florida, Tampa, FL 33620

Email: Amin Ahmadi Adl - amin1@mail.usf.edu; Xiaoning Qian*- xqian@ece.tamu.edu; Ping Xu - Ping.Xu@epi.usf.edu ; Kendra Vehik - Kendra.Vehik@epi.usf.edu; Jeffrey P. Krischer - Jeffrey.Krischer@epi.usf.edu;

*Corresponding author

1 Bio-marker identification in DPT-1 based on treated subjects

As mentioned in the paper, the subjects under study in both "high risk" and "intermediate risk" groups were randomly divided into two roughly equal sub-groups: one received parenteral or oral insulin supplement while the other was assigned to the placebo arm of the corresponding trial [1–3]. We consider the treated subgroups of both "high risk" and "intermediate risk" groups as a new dataset for our data-driven analysis. This new dataset contains 357 subjects within which 124 subjects developed T1D at the end of the study. We check the performance of both individual-based and network-based bio-marker identification methods using the "embedded" cross-validation procedure described in the paper. The results are shown in Table S1. As one can see, the AUC obtained by our network-based feature ranking is significantly (with p-values < 0.002) higher than individual-based feature ranking but we have only a marginal improvement based on the estimated prediction accuracy. The final set of bio-markers are selected as the features that at least appear in 40% of the 1000 (i.e. 400) selected subsets during our "embedded" cross-validation. We also perform an exhaustive search to find the best possible set of bio-markers for this new dataset. The bio-markers obtained by individual-based method, network-based method, and exhaustive search together with their corresponding measured performances (based on 100 repeated ten-fold cross-validation) are shown in Table S2. We further provide the intersection of the bio-markers identified using different methods in Figure

S1. As one can see, the intersection of the bio-marker set obtained by our network-based method and the best set of bio-markers (by exhaustive search) is larger than the intersection of bio-markers identified by individual-based method and the best set of bio-markers. This shows that the bio-marker set identified by our network-based method is closer to the best possible set of bio-markers. Also, the average of 1000 synergy networks obtained in our "embedded" cross-validation procedure is shown in Figure S2. Comparing this network with the network in Figure 4 of the paper reveals that the treatment perturbation may change the synergistic effect of the features on disease outcome. Consequently, a different set of bio-markers are identified for treated subjects compared to the bio-markers identified for untreated group.

Performance measure	Individual ranking	Network-based ranking	p-value
Accuracy	67.28%	67.50%	0.42
AUC	0.6224	0.6324	0.0016

Table S1: Comparing the accuracy and AUC performance of the network-based spectral feature ranking with individual based feature ranking based on the Treated group in DPT-1 dataset.

Performance Measure	Individual ranking		Network-based ranking		Exhaustive search	
Accuracy	Height; 2-h glucose; ICA; HOMAIR; Peak C-Peptide; FPIR-to-HOMA-IR ratio;	69.88%	Weight; 2-h glucose; ICA; FPIR; Fasting insulin (IVGTT); HOMAIR; Peak C-Peptide; AUC C-Peptide;	69.33%	2-h glucose; Age; BMI; FPIR-to-HOMA-IR ratio; Fasting glucose (IVGTT); Fasting insulin (IVGTT); HOMAIR; ICA; Weight;	72.89%
AUC	Height; ICA; FPIR-to-HOMA-IR ratio;	0.6434	2-h glucose; ICA; FPIR; Fasting insulin (IVGTT); HbA1c; Peak C-Peptide;	0.6654	2-h glucose; BMI; FPIR; Fasting insulin (IVGTT); GAD; ICA; Peak C-Peptide; Weight;	0.7028

Table S2: Final sets of bio-markers by optimizing accuracy and AUC for the Treated group in DPT-1 dataset.

Checking the effect of λ on the performance by spectral feature ranking

Based on the formulation in Equation (1) in the manuscript, for λ values close to zero, the interactive effects are neglected in feature ranking. The results from our spectral ranking based on estimated synergy networks will be very similar to individual-based feature ranking. On the other hand, for large values of λ, the interactive effects between variables become dominant for feature selection. In our current implementation, we take $\lambda = 1.0$, which assumes equal importance for both individual and interactive effects. However, with different λ values, we might get different feature rankings. To illustrate the sensitivity of feature ranking

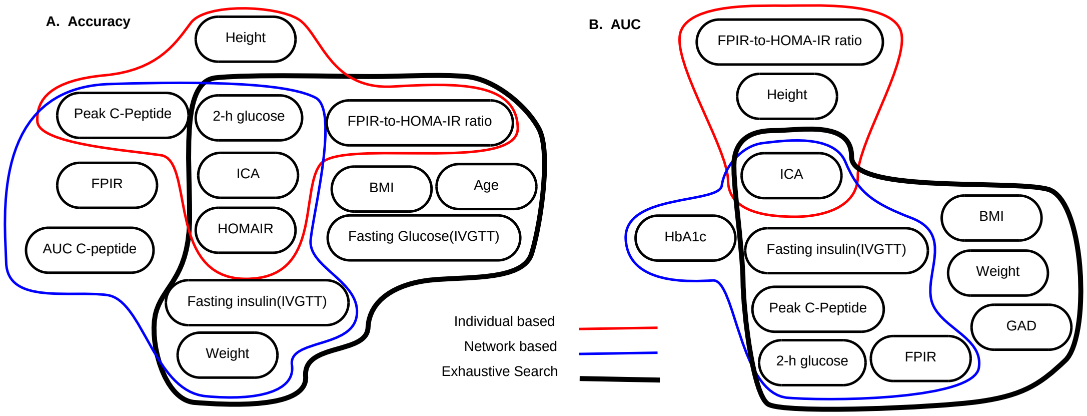

Figure S1: Venn diagrams illustrating the Bio-markers identified for the Treated group in DPT-1 dataset using different methods. **A:** Bio-markers identified by optimizing Accuracy. **B:** Bio-markers identified by optimizing AUC.

with respect to different λ values, we have estimated prediction accuracy using our second set of simulated datasets (exactly as explained in the manuscript) for the following nine different values of λ: 0.1, 0.3, 0.5, 1.0, 1.5, 2.0, 3.0, 5.0, 10.0 and the trend is illustrated in Figure S3 It is clear that for small λ values, the accuracy is similar to individual-based ranking which is 60.38% while for values close to 1.0, the performance is stable and very close to 65.47% obtained by setting $\lambda = 1.0$ as in the manuscript.

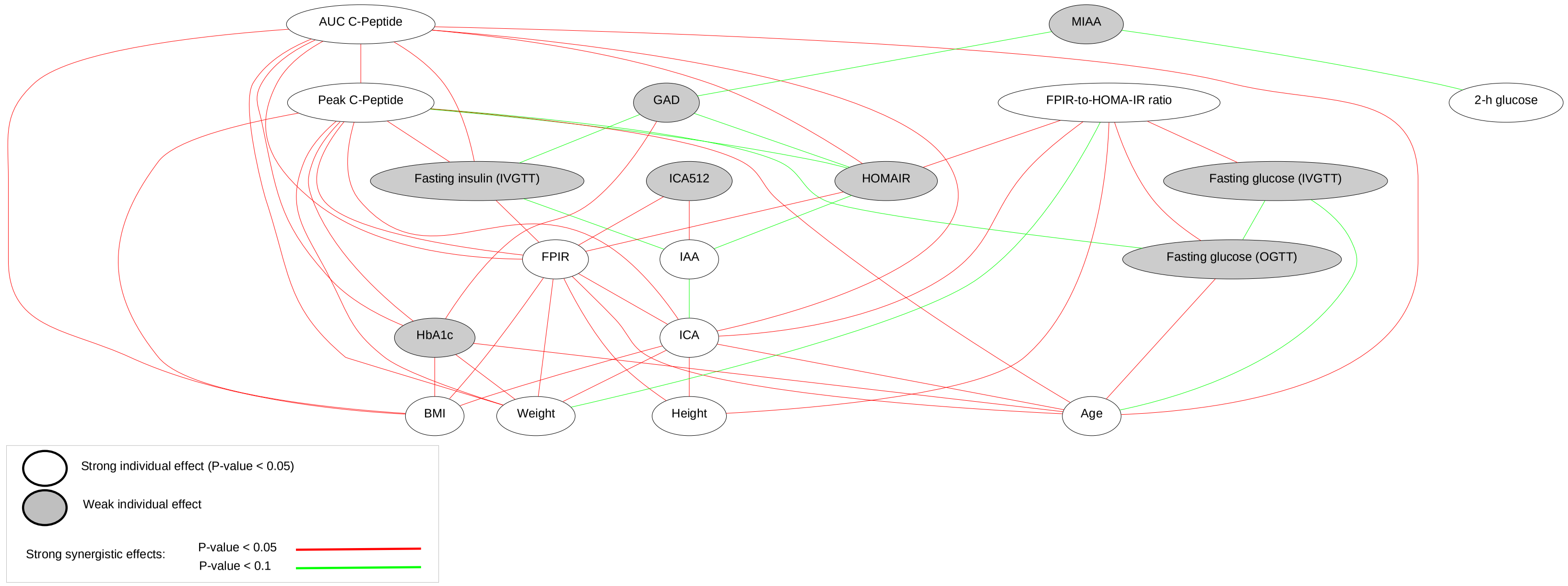

Figure S2: The average synergy network for the Treated group in the DPT-1 dataset.

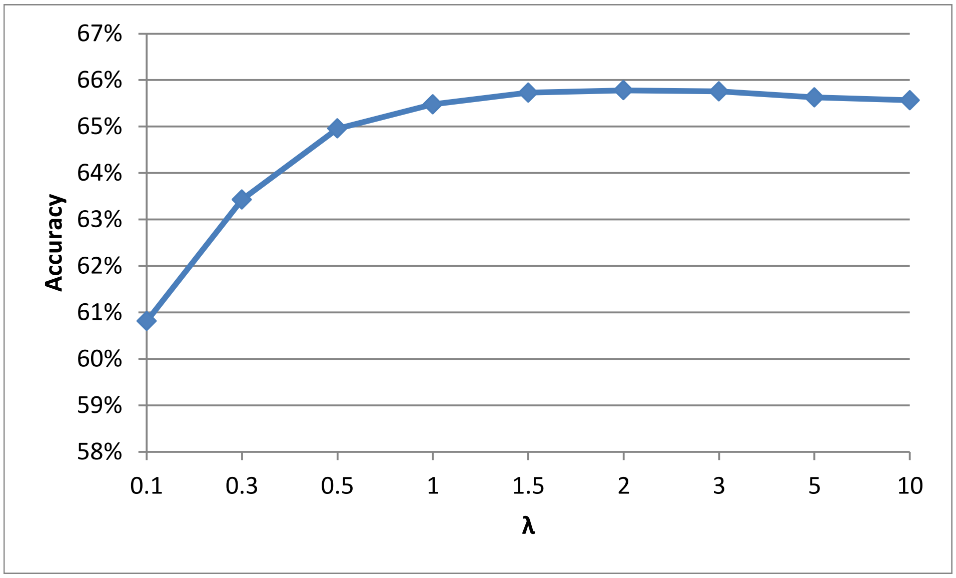

Figure S3: Plot showing the effect of λ on the performance of the bio-marker identification based on simulated datasets.

References

1. Krischer J, Cuthbertson D, Yu L, Orban T, Maclaren N, Jackson R, Winter W, DA DS, Palmer J, GS GE: **Screening strategies for identification of multiple antibody-positive relatives of individuals with type 1 diabetes**. *J Clin Endocrinol Metab* 2003, **88**:103–108.

2. Sosenko J, Palmer J, Greenbaum C, Mahon J, Cowie C, Krischer J, Chase H, White N, Buckingham B, Herold K, Cuthbertson D, Skyler J, the Diabetes Prevention Trial-Type 1 Study Group: **Increasing the accuracy of oral glucose tolerance testing and extending its application to individuals with normal glucose tolerance for the prediction of type 1 diabetes**. *Diabetes Care* 2007, **30**:38–42.

3. Xu P, Wu Y, Zhu Y, Dagne G, Johnson G, Cuthbertson D, Krischer J, Sosenko J, Skyler J, the DPT-1 Study Group: **Prognostic Performance of Metabolic Indexes in Predicting Onset of Type 1 Diabetes**. *Diabetes Care* 2010, **in press**(doi: 10.2337/dc10-0802).
